# Species partitioning in a temperate mountain chain: Segregation by habitat vs. interspecific competition

**DOI:** 10.1002/ece3.2883

**Published:** 2017-03-19

**Authors:** Giulia Bastianelli, Brendan A. Wintle, Elizabeth H. Martin, Javier Seoane, Paola Laiolo

**Affiliations:** ^1^ Research Unit of Biodiversity (UO, CSIC, PA)Universidad de OviedoMieresSpain; ^2^ School of BioSciencesThe University of MelbourneParkvilleVic.Australia; ^3^ Terrestrial Ecology GroupDepartamento de EcologíaUniversidad Autónoma de MadridMadridSpain

**Keywords:** geographical zonation, interspecific interference, joint species distribution modelling, passerines, territorial intrusion experiments

## Abstract

Disentangling the relative influence of the environment and biotic interactions in determining species coexistence patterns is a major challenge in ecology. The zonation occurring along elevation gradients, or at bioclimatic contact zones, offers a good opportunity to improve such understanding because the small scale at which the partitioning occurs facilitates inference based on experiments and ecological modelling. We studied the influence of abiotic gradients, habitat types, and interspecific competition in determining the spatial turnover between two pipit and two bunting species in NW Spain. We explored two independent lines of evidence to draw inference about the relative importance of environment and biotic interactions in driving range partitioning along elevation, latitude, and longitude. We combined occurrence data with environmental data to develop joint species distribution models (JSDM), in order to attribute co‐occurrence (or exclusion) to shared (or divergent) environmental responses and to interactions (attraction or exclusion). In the same region, we tested for interference competition by means of playback experiments in the contact zone. The JSDMs highlighted different responses for the two species pairs, although we did not find direct evidence of interspecific aggressiveness in our playback experiments. In pipits, partitioning was explained by divergent climate and habitat requirements and also by the negative correlations between species not explained by the environment. This significant residual correlation may reflect forms of competition others than direct interference, although we could not completely exclude the influence of unmeasured environmental predictors. When bunting species co‐occurred, it was because of shared habitat preferences, and a possible limitation to dispersal might cause their partitioning. Our results indicate that no single mechanism dominates in driving the distribution of our study species, but rather distributions are determined by the combination of many small forces including biotic and abiotic determinants of niche, whose relative strengths varied among species.

## 1 Introduction

Aspects of the ecological niche shape species geographic distribution and co‐occurrence patterns (Holt & Keitt, 2005). Environmental or abiotic factors, such as climatic and topographic conditions, directly influence the distributions of species by filtering them on the basis of their physiological tolerances (Dunson & Travis, 1991). They act also indirectly by generating patterns in seasonality and productivity, which influence population density and regional species richness (Kissling, Field, & Böhning‐Gaese, 2008). Biotic interactions also influence the ability of species to settle in certain environments and to co‐exist (Case & Taper, 2000).

Among interspecific interactions, competition is one of the most relevant and it may occur via two mechanisms, resource exploitation and interference (Amarasekare, 2002; Case, Holt, McPeek, & Keitt, 2005). Through resource exploitation, the common form of competition between animals, interacting species influence each other by directly consuming and reducing a limited resource (Vance, 1984). Conversely, interference competition consists in negative direct interactions between two species mediated by territoriality and despotic behavior (e.g., Jankowski, Robinson, & Levey, 2010) which then limit their ability to use a shared resource (Schoener, 1983). Interference involves the development of costly competition traits and becomes beneficial only if species overlap broadly in resource use (Losin, Drury, Peiman, Storch, & Grether, 2016; Orians & Willson, 1964). Competition ultimately leads to the segregation and competitive exclusion of subordinate species in any given place (Robinson & Terborgh, 1995). Thus, the global distribution of species is driven by complex interactions between current ecological influences (environmental factors and biotic interactions), evolutionary history, environment‐specific limitations on dispersal and reproductive strategies, making the study of geographic range drivers a challenging but exciting ecological research priority (Sexton, McIntyre, Angert, & Rice, 2009).

The relative importance of environmental factors and competitive interactions in shaping species distribution and promoting coexistence may vary with the environmental and geographic context and scale (Brown, Stevens, & Kaufman, 1996; Sexton et al., 2009). In general, negative interactions become less important in more stressful environmental conditions, in keeping with the Stress Gradient Hypothesis (Barrio, Hik, Bueno, & Cahill, 2013). At high elevations, high latitudes, or in extremely dry environments, competitive interactions tend to weaken because harsh conditions reduce population numbers, and thus the opportunities for negative interactions, as well as the energy available for costly defenses or competition traits (Barrio et al., 2013; Brown et al., 1996). This process has received the greatest attention in plant ecology (e.g., Callaway et al., 2002) but may also explain why, in tropical fauna, negative biotic interactions have been documented more frequently than in temperate assemblages (Schemske, Mittelbach, Cornell, Sobel, & Roy, 2009). Several studies on animals showed that competition mediates the elevational partitioning in tropical mountains (e.g., Cadena & Loiselle, 2007; Jankowski et al., 2010; Pasch, Bolker, & Phelps, 2013). In temperate mountains, where conditions are harsher and more seasonal, fewer studies investigated the role of competition in faunal elevational partitioning. These found evidence of both biotic and abiotic influences roles but the latter appears to be stronger (Elsen, Tingley, Kalyanaraman, Ramesh, & Wilcove, 2017; Freeman & Montgomery, 2015; Noon, 1981).

Recent advances in species distribution modeling, particularly joint species distribution modeling (JSDM—Ovaskainen, Hottola, & Siitonen, 2010; Pollock et al., 2014; Royan et al., 2016), has improved our capacity to disentangle the respective roles of environmental factors and biotic interactions in shaping species distributions and co‐occurrence patterns. Joint species distribution modeling combines species distribution modeling (Elith & Leathwick, 2009) with species co‐occurrences, and permits estimation of the relative contribution of environmental drivers and biotic interactions on observed co‐occurrence patterns, provided all the important predictors of the modeled species are considered (Pollock et al., 2014; Royan et al., 2016). However, these models are restricted to inference based on correlation and do not provide a test of causation; for this purpose, experimentation is required. Experimental work involving removal is often unfeasible or ethically questionable in animal assemblages. However, for species that use conspicuous behaviors to advertise and defend territory, detection of aggressive behavioral interference leading to segregation may support identification of causal mechanisms (Laiolo, 2012, 2013). Such experiments involve the observation of behavioral responses to a simulated territorial intrusion, usually triggered by acoustic signals or decoys (e.g., Jankowski et al., 2010).

In this study, we combined spatial, multispecies modeling and experimental approaches to investigate the roles of interspecific competition and environmental factors in determining the range limits of closely related birds in the Cantabrian Mountains (NW of Spain). We focused our study on a sympatric species pool in which closely related species (i.e., belonging to the same genus) co‐occur at the regional scale but show fine‐scale partitioning. We focussed on two pairs of congeneric passerines: the Tree pipit (*Anthus trivialis*) and the Water pipit (*A. spinoletta*), and the Yellowhammer (*Emberiza citrinella*) and the Ortolan bunting (*E. hortulana*). We aimed at testing for the effect of biotic interactions in the distribution of these birds along geographical gradients. To our knowledge, this kind of approach has not been previously applied to the context of European mountains, and the role of biotic interactions in determining faunal zonation in European mountains is still poor known. We utilize ecological modeling and experimental approaches to address two specific questions: (1) to what extent does the environment and congener presence appear to influence the observed occupancy (and co‐occupancy) data for the two pairs of congeners; and (2) is interspecific interference competition between congeners evident in behavioral responses of the pairs of species in their overlap zone? To address the first question, we quantified the spatial segregation in each congeneric pair and then developed a JSDM for each species pair in order to quantify both environmental and residual correlations (i.e., potentially due to interactions) between congeneric species, providing inference about the relative importance of environmental and potential behavioral influences on the ranges of both pairs of species. To address the second question, we simulated interspecific territorial intrusions by means of playback experiments in the overlapping areas. Based on existing hypotheses, we expected to observe that competition plays a weak role in shaping the distribution of these congeneric species in the environmentally stressful, seasonal montane, and alpine conditions of our study area (Barrio et al., 2013; Meléndez et al., 2014). Consistently with this expectation, we should observe (1) a high segregation level at local scale but (2) null model residual correlations and (3) a stronger response to conspecific than heterospecific playbacks, if local abiotic processes predominate in determining the spatial distribution of the species. Shared environmental correlations should be strong and they would vary from positive to negative, depending on whether species occupy similar environmental conditions because of common ancestry or have instead diverged in some aspects of their niche (because of character displacement or in response to differential selection pressures within their respective ranges). Otherwise, we expected (4) a negative residual correlation in models (i.e., species distribution conditioned by the occurrence of congeners) and (5) heterospecific aggressiveness emerging from experiments, if current ecological processes in the form of interference competition are more relevant in shaping the distribution of the species (Jankowski et al., 2010; Pasch et al., 2013).

## 2 Materials and Methods

### 2.1 Data collection

#### 2.1.1 Study area and species

**Figure 1:**
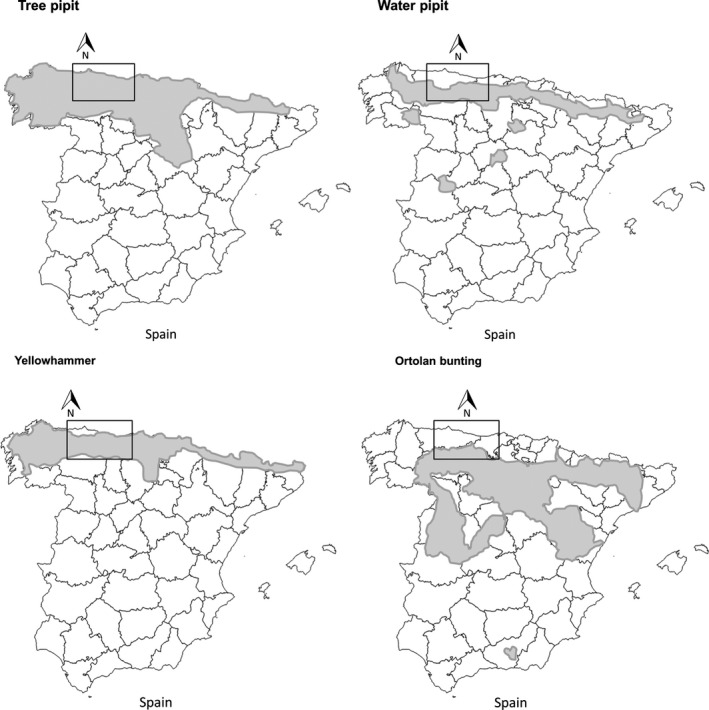
Distribution of the Tree pipit, Water pipit, Yellowhammer, and Ortolan bunting in Spain. The shaded areas depict the distributions of pipits and buntings species. Modified from Martí and Del Moral (2003). Atlas de las Aves Reproductoras de España. Dirección General de Conservación de la Naturaleza‐Sociedad Española de Ornitología. Madrid. The rectangles enclose the study area for species' survey; the experiment was performed in the contact zone only

The study was carried out in the Cantabrian Mountains, a mountainous area 500 km long from the easternmost to the westernmost fringes, 120 km wide in the north–south direction, and 2648 m a.s.l. high (Figure 1; Appendix S1). The climate can be classified as humid Atlantic in the north, alpine in the highlands, and oro‐Mediterranean in the south. The average annual temperature ranges from 1.9 to 13.6°C and the annual rainfall from 482 to 2,129 mm. The habitat is characterized by deciduous forests, shrubberies, grasslands, and rocks. The treeline is found between 1,000 and 1,600 m a.s.l. and pseudo‐alpine grasslands are common because of historical clearing and grazing by domestic livestock (García, Quevedo, Obeso, & Abajo, 2005).

Tree pipit, Water pipit, Yellowhammer, and Ortolan bunting inhabit montane, alpine, and subalpine open habitats in our study area and present relatively overlapping trophic niches, being pipits more strictly insectivorous and buntings granivorous outside the breeding period (Brodmann, Reyer, Bollmann, Schläpfer, & Rauter, 1997; Dale & Manceau, 2003; Loske, 1987). Tree pipit and Ortolan bunting are trans‐Saharan migrants (Dale & Manceau, 2003; Loske, 1987). All species are territorial, mostly monogamous, and they nest on the ground. They actively defend territories in the breeding period, and males sing to mark territories and attract females. These species served as models in studies on homo‐ or heterospecific territoriality, as they reliably respond to playbacks simulating territorial intrusion (Bastianelli, Seoane, Álvarez‐Blanco, & Laiolo, 2015; Osiejuk, Ratyńska, & Cygan, 2004; Petrusková et al., 2014; Skierczynski, Czarnecka, & Osiejuk, 2007).

In Spain, the Tree pipit is distributed along Euro‐Siberian and supra‐Mediterranean regions from the coast up to mountainous slopes (Purroy, 2003; Figure 1). Its congener Water pipit also occupies the Euro‐Siberian region and some areas of the central system but systematically above 700 m a.s.l. (Vasquez, 2003). These species show therefore a noticeable elevational partitioning. In the Cantabrian Mountains, the Tree pipit reproduces in low‐ and medium‐elevation grasslands (average elevation ± SD: 1230.39 ± 416.60 m a.s.l.). Conversely, the Water pipit reproduces in medium and high elevations (average elevation ± SD: 1726.63 ± 341.35 m a.s.l.; Figure 1; Laiolo et al., 2015; Meléndez & Laiolo, 2014).

The Yellowhammer is distributed in the Euro‐Siberian and part of the northern supra‐Mediterranean regions of Spain (Figure 1), in mountainous areas above 800 m a.s.l. (Arratibel, 2003). The Ortolan bunting is distributed in northern Spain but is absent from the northernmost Euro‐Siberian (Atlantic) region (Figure 1) and is found only in the southern Cantabrian Mountains (Pons, 2003). Bunting species show both latitudinal and longitudinal partitioning. The Yellowhammer is found at mid‐elevations throughout the study area (average elevation ± SD: 1335.65 ± 295.19 m a.s.l.), while the Ortolan bunting only is present in the southwestern slopes but at roughly similar elevations (average elevation ± SD: 1590.62 ± 204.98 m a.s.l.; Laiolo et al., 2015).

The replacement areas of both species pairs consist of grasslands interposed with scattered trees, shrubs, and crops between 700 and 1,800 m a.s.l.

#### 2.1.2 Bird surveys, environmental predictors, and qualitative estimation of local segregation

During the breeding periods of 2009–2014, we surveyed the bird community of the Cantabrian Mountains from 120 to 2,620 m a.s.l. over a land area of 16,000 km^2^ (Appendix S1). Birds were surveyed in 2,346 circular plots of 100 m radius, separated by 400 m from each other. These plots were arranged along 5–24 km transects. In order to track the breeding phenology along the elevation gradient, we commenced fieldwork at the end of March (when migrants arrive) in lowlands and we finished in July in the highlands. Plots were surveyed from sunrise until midday in good weather conditions (Bibby, 2000). Each plot was visited only once (for details see Laiolo et al., 2015).

In each plot, we estimated a suite of continuous environmental variables that commonly influence bird distributions, with particular focus on those variables with a known influence on the study bird species. Climatic, topographical, local habitat, and geographical variables were all considered. Climatic variables could influence the species distribution fundamental ecological niche due to physiological constraints and/or the food availability (Chamberlain, Brambilla, Caprio, Pedrini, & Rolando, 2016; Meléndez & Laiolo, 2014). We estimated the annual averages for the mean, maximum and minimum temperatures, the average annual rainfall, the average temperature range, and accumulated precipitation (difference between maximum and minimum annual precipitation and temperatures) in a buffer of 100 m around the center of the plot. Topographical variables may influence the presence of suitable nesting sites as well as food availability. We extracted the average slope (measured in degree and extracted from a digital elevation model grid) and the mean solar radiation (kJ m^−^² day^−1^, potential radiation input reaching the soil in standard and uniform atmospheric conditions) in a buffer of 100 m around the center of the plot, and also an index of roughness (calculated as a difference between the minimum and maximum elevation of each plot). Climate and solar radiation were inferred from the digital layers of the Climate Atlas of the Iberian Peninsula. GIS layers representing each variable were built with a resolution of 200 m by modeling 15 years of meteorological data from local stations of the Spanish National Meteorological Institute (Ninyerola, Pons, & Roure, 2005). Microhabitat categories and microhabitat structure capture the broad characteristics of species' niche, from the distribution of food, nest site, and shelter to their availability, quality, and quantity (Dale & Manceau, 2003; Meléndez & Laiolo, 2014). We estimated *in situ* the percent cover of five microhabitat categories within 100‐m circles centered on sampling points: grassland (all grassland and herbaceous species), high shrub (>1 m), low shrub (<1 m), forest, rock, and bare ground (e.g., Laiolo et al., 2015; Meléndez & Laiolo, 2014). We calculated an index of microhabitat heterogeneity from these microhabitat proportions by means of the Simpson index. This index ranges from 0 in homogeneous habitats (one habitat type) to 1, when all types of habitat are equally represented (Simpson, 1949). Geographical variables are surrogates for temperature and/or precipitation variability and may indicate the existence of barriers to dispersion (Chamberlain et al., 2016). We established latitude, longitude, and elevation of the center of each plot by means of a GPS.

We quantified spatial segregation in each congeneric pair estimating the checkerboard score (C‐score; Stone & Roberts, 1990) at local scale (in Cantabrian Mountains) and also at wide geographical scale (in Europe). At the local scale, we used occurrence data obtained from our bird surveys. At European scale, we used presence/absence data downloaded from the Atlas of European Breeding Birds in 2,500 km² square cells (Hagemeijer & Blair, 1997; http://ipt.sovon.nl/). The checkerboard score varies between 0 (complete sympatry) and 1 (complete segregation) (Stone & Roberts, 1990). The C‐score was calculated by means of R package “bipartite” (v3.2.2; Dormann, Gruber, & Fründ, 2008).

### 2.2 Statistical analysis

#### 2.2.1 JSDM analysis of species distributions and co‐occurrence

Joint species distribution modeling (JSDM) is a statistical approach that decomposes species co‐occurrence patterns into two components: shared environmental response and residual co‐occurrence (Pollock et al., 2014). In our species‐pair system, the former reflects the correlated responses of species to the habitat, topography, and climate variables (positive: similar response, negative: diverging response). The latter represents the correlation between species occurrences, after controlling for their shared environmental preferences (positive for co‐occurrence, negative for exclusion or for other ecological processes entailing a negative correlation). Joint species distribution modeling uses Bayesian probit multivariate regression to estimate the probability of co‐occurrence as a function of predictors (details on this procedure can be found in Pollock et al., 2014 and Royan et al., 2016).

The JSDM estimates the posterior distributions for three types of parameters: regression coefficients for each species environmental predictor, correlation between species due to the environment, and residual correlation between species occurrence (Pollock et al., 2014). A significant environmental correlation (i.e., the 95% credible intervals do not cross 0) indicates shared or divergent environmental preferences. On the other hand, a significant residual correlation indicates contribution of interspecific interaction. Together, these two correlations allow interpretation of whether co‐occurrences are driven primarily by environmental or competitive process, or both. Nevertheless, the absence of unmodelled environmental predictors leads to spurious significant residual correlations (Pollock et al., 2014).

We used presence/absence survey dataset but excluded survey plots characterized by high forest cover, as such surveyed areas were unsuitable and, consequently, they would contain no useful information for the modelling. For pipits, we considered only survey plots where the percent tree cover is less than 80 % of the area (*N* = 1,874 plots), because the Tree pipit is an ecotone species that utilizes a mixture of open grasslands and scattered trees (Laiolo, Dondero, Ciliento, & Rolando, 2004). For buntings, we selected survey plots where the tree percent cover is <60% of the area of the plot (*N* = 1,790 plots), being both species less dependent on tree cover (Dale & Manceau, 2003). Our sample size corresponds to 192 presences for the Tree pipit, 655 presences for the Water pipit, 161 presences for the Yellowhammer, and 52 presences for the Ortolan bunting.

We developed a set of alternative JSDMs for each pair of congeneric species, considering a different set of combinations of environmental variables representing drivers (or surrogates of drivers) for pipits and bunting. In order to build realistic set of alternative models and to avoid overfitting problems, we filtered for the most important environmental predictors for each species pair among all the predictors measured in the survey plots using a documented variable screening approach (Appendix S2). We considered quadratic effects for mean annual temperature, high shrub, and rock covers in pipits (Appendix S2). For buntings, which have a smaller number of observations, we considered only linear predictors to avoid overparametrization and nonconvergence of models (Appendix S2). All predictors were centered and scaled by their standard deviations.

Finally, we carried out a cross‐validation analysis in order to evaluate the predictive capability of alternative JSDMs and to select those with best performance. For this, we performed *K*‐fold cross‐validation by randomly splitting the dataset in *k *=* *5 equal‐sized subsets that maintained the overall proportional prevalence of presences and absences in each fold. The average (across the *k* folds) area under the curve (AUC) and the corresponding standard deviation were obtained to identify the best candidate model in predicting the presence and absence of the species.

We performed the JSDMs and cross‐validation analysis by means of Markov Chain Monte Carlo Bayesian software JAGS v3.4.0 in R v3.2.2 (R Development Core Team, 2015) via R2jags v0.03‐11 (Plummer, 2013). We run three chains for 150,000 iterations for pipits (first 75,000 discarded as burn‐in and the remaining samples thinned by a factor of 75) and 250,000 iterations for buntings (first 125,000 discarded as burn‐in and the remaining samples thinned by a factor of 125). Model convergence was visually checked using diagnostic plots (density and trace plots). Vague normal priors were used to model parameters (mean = 0; precision = 0.001). All model fitting and evaluation codes are provided in Appendices [Link], [Link], [Link].

#### 2.2.2 Playback experiment design

We performed playback experiments simulating territorial intrusion in replacement areas, that is where congeners were located ≤2 km from each other (Appendix S1). The study was performed during breeding, which is the sole phase of species phenology in which birds are strongly territorial and in which their ranges overlap (one member of each pair is migratory and spends the winter elsewhere). During playbacks, we broadcast the songs of a conspecific male, or a congeneric male, or of a control species. Overall, we tested 148 pipit males and 112 bunting males; each individual was tested once and was randomly submitted to a playback of one of three categories mentioned above (conspecific, congener, or control; Appendix S6). We selected as controls species of a different family and that largely co‐occurred with the target species, which we assumed were no competitors. Yellowhammer and Whinchat (*Saxicola rubetra*) were selected as the control species for pipits and buntings, respectively. Similar to other playback studies, we considered that interspecific territoriality occurred if the behavioral response did not differ between conspecific and congeneric playbacks and/or if the response to the congeneric playback was stronger than to the control playback (Jankowski et al., 2010; Laiolo, 2013).

Songs used for the playbacks were recorded from individuals of each species from the end of March to July of 2012, 2013, and 2014 in the Cantabrian Mountains. Playback stimuli were created using Avisoft‐SasLab Pro (Version 3.91) Software by Raimund Specht (Berlin, Germany; Appendix S6). We presented each recorded song as playback stimulus to only one individual per species following recommendations by Kroodsma, Byers, Goodale, Johnson, and Liu (2001). Experiments were performed during the breeding season in the end of March, April, May, June, and July from 07:00 to 16:00 hr in the replacement areas during the same years. The tested males were located by means of mapping individual territories, and after surveying their activity to be sure, they sang and displayed within them (Bastianelli et al., 2015). Each experiment lasted 12 min: 4 min of silence, where the focal individual was observed in the absence of stimuli (preplayback period), followed by 4 min of conspecific, congener, or control playback broadcast (playback period), and then by 4 min of silence again (postplayback period). Three behavioral variables were measured as indices of territoriality from the start of the playback period to the end of the postplayback period: the minimum distance of approach (m) to the speaker, the time (s) of the closest approach (latency of approach), and the number of songs emitted (Appendix S6; Bastianelli et al., 2015; Laiolo, 2013). In order to confirm the ability of playback experiments in stimulating the target species, and to identify which behavioral response was involved in territorial defense, we initially compared bird behavior in the preplayback vs. playback/postplayback periods during the simulated intrusion of a conspecific in each studied species. The individuals of each species reached closer distances to the speaker during and after the playback experiment than during the preplayback period (sign tests: all *z* ≥ 2.46, all *p* ≤ .01). Thus, we considered the closest approach distance and the latency of approach as reliable proxies of territorial behaviors for all the study species. However, we excluded song rate because we did not detect a change in song activity between pre‐experiment observation and playback and postplayback observation (all *p* > .60, all *z* ≤ 0.55). We assumed that if we could not detect a change in acoustic response to a conspecific territorial intrusion, it was unlikely that we could observe such change as a reaction to an interspecific intrusion.

#### 2.2.3 Analysis of playback experiments

We performed a preliminary analysis to test whether the month, the time of the day in which a test was performed, and their interaction could affect bird behavioral patterns. In no species we found such effects on the closest distance of approach or on the latency of the approach (linear models: all *p* ≥ .10). Therefore, we did not account for temporal covariates in further analyses. In order to assess the differences in the minimum distance of approach between the three playback levels (conspecific, congeneric, and control song), we performed a one‐way analysis of variance (ANOVA) after transforming the variable by means of a Box‐Cox transformation to meet the assumptions of normality (Tree pipit: λ = 0.30, Water pipit: λ = 0.26, Yellowhammer: λ = 0.30; Ortolan bunting: λ = 0.51). We performed multiple comparisons (Tukey contrasts) to assess the significance of the differences between pairwise playback types. As the latency of approach did not meet the normality assumption, we carried a Kruskal–Wallis test to analyze playback effects. We performed multiple comparisons by means of Dunn test. We performed power tests in the case of detecting no significant differences in the behavioral response between pairwise playback types, and we based our expectations of interference on the local spatial segregation patterns (C‐score) observed in each species pairs. A power ≥0.80 was considered as a good power (Cohen, 1992). We performed the analysis with R v 3.2.2 (R Development Core Team, 2015) and G power v. 3 (Faul, Erdfelder, Lang, & Buchner, 2007).

## 3 Results

Congeners segregated locally, but are sympatric when considering their European distribution. The C‐score at the local scale is 0.90 for pipits and 0.71 for buntings (1 is the maximum threshold for this index, which indicates full segregation). However, complementarity at the scale of the European continent is lower. It drops to 0.07 for pipits and 0.09 for buntings, being very close to the minimum value (0) of complete sympatry, indicting largely shared distributions at the continental scale.

### 3.1 JSDM analysis of distribution and co‐occurrence

**Figure 2:**
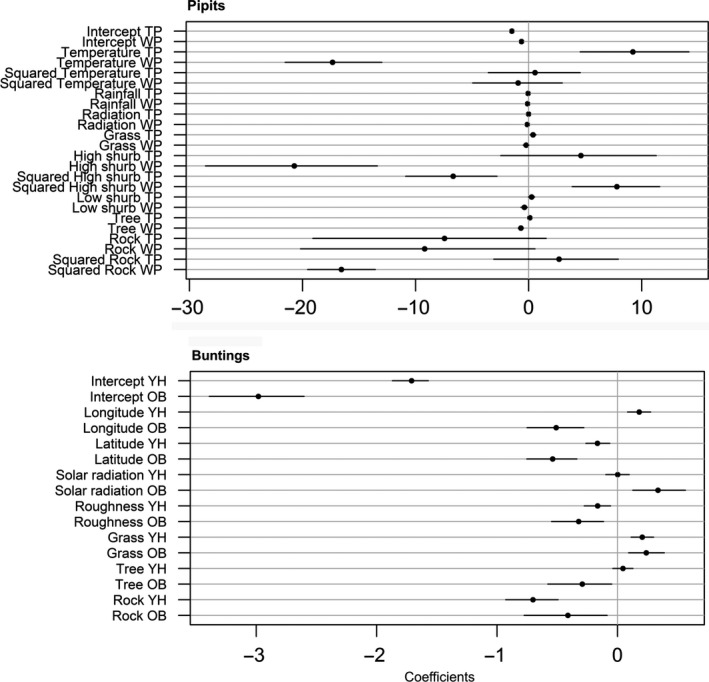
Plots representing the highest posterior density mean of the coefficients (intercepts and slopes), their lower (2.5%) and the upper (97.5%) credible intervals, for of JSDM with the highest AUC in pipits and buntings (TP = Tree pipit; WP = Water pipit; YH = Yellowhammer; OB = Ortolan bunting)

**Figure 3:**
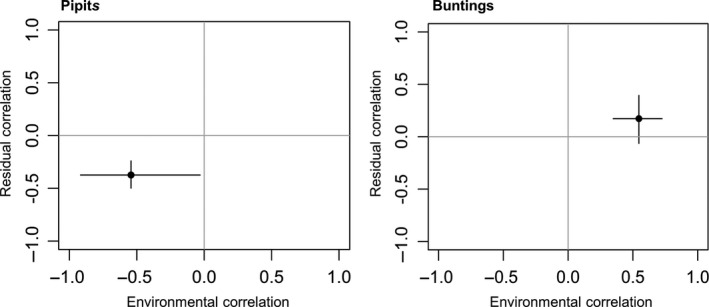
Highest posterior density means of environmental and residual correlations, and their lower (2.5%) and the upper (97.5%) credible intervals, estimated in JSDM with the highest AUC in pipits and buntings

For pipits, the best JSDM, as measured by predictive ability, included climatic, geographical, topographical, and habitat predictors for both species (Figure 2); habitat predictors increased markedly the predictive power of the model for both species (Appendix S7). The AUC of the best JSDM for pipits was 0.75 for the Tree pipit and 0.83 for the Water pipit, representing a good to very good predictive discrimination between occupied and unoccupied sites. The range of shared environmental correlations was negative for pipits, thus suggesting that species had different environmental requirements (Figure 3). In the Tree pipit, the probability of presence increased with temperature, grassland, low shrub, and tree covers and at intermediate percentages of high shrubs (Figure 2). In the Water pipit, conversely, the probability of presence decreased with temperatures and tree cover and at intermediate percentages of high shrubs, but it increased at intermediate percentages of rocks (Figure 2). Apart of their negative environmental correlation, pipits also co‐occurred less than expected given their response to environmental predictors. The residual correlation was negative and significant (~ −0.5 with 95% credible intervals excluding zero; Figure 3).

For buntings, the best predictive JSDM included geographical, topographical, and habitat predictors (Figure 2; Appendix S7). The predictive power of the model including climatic predictors was lower than geo‐topographic variables in the Ortolan bunting, and lower than the model including habitat variables in the Yellowhammer (Appendix S7). The AUC of the best JSDMs was 0.80 for Yellowhammer and 0.91 for Ortolan bunting, representing very good predictive discriminations between occupied and unoccupied sites. Buntings showed a positive association due to shared environmental responses (Figure 3, Appendix S7). They were more common at the southern plots, although this tendency was stronger in the Ortolan bunting. Both species selected flat places with high grassland and low rock cover (Figure 2). However, the probability of presence of the Yellowhammer increased eastwards while that of the Ortolan bunting increased westwards (Figure 2). Furthermore, the Ortolan bunting was more frequent in areas characterized by high solar radiation and low tree cover (Figure 2). The residual correlation was positive but the estimate was uncertain; thus, there is not really any convincing evidence of positive interactions (Figure 3).

### 3.2 Playback experiments

**Figure 4:**
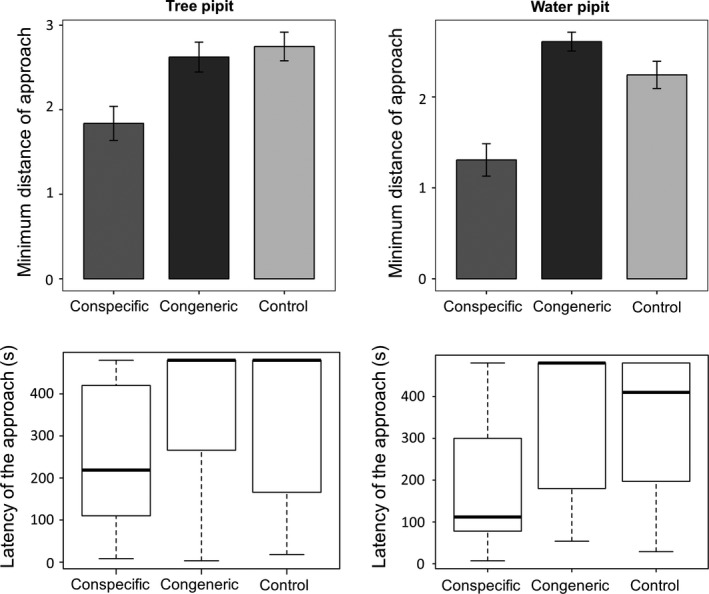
Behavioral responses to playback experiments in pipits. In top plots, the minimum distances of approach (after Box‐Cox transformation) during conspecific (dark gray), congeneric (black), and control (pale gray) trials are shown. Bars show the means ± SE. In bottom plots, the latencies of approach during conspecific (dark gray), congeneric (black), and control (pale gray) trials are shown. For each box plot, the total data range, the 25% and 75% quartiles (box), and the median are represented

**Figure 5:**
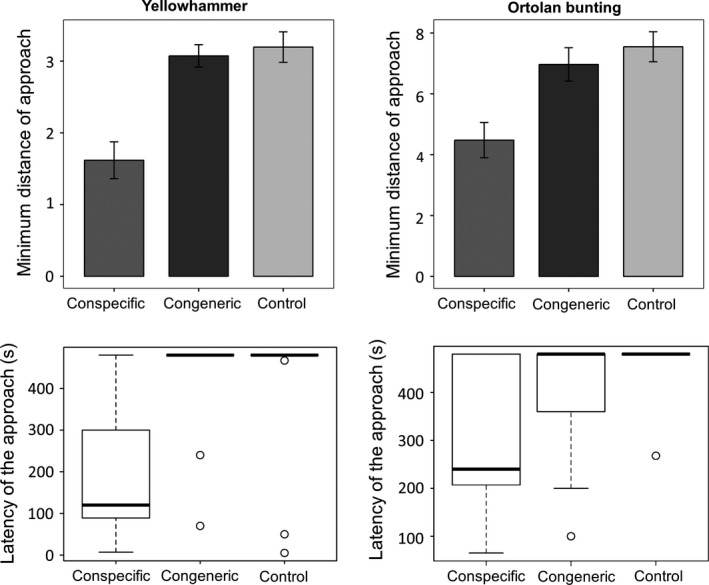
Behavioral responses to playback experiments in buntings. In top plots, the minimum distances of approach (after Box‐Cox transformation) during conspecific (dark gray), congeneric (black), and control (pale gray) trials are shown. Bars show the means ± SE. In bottom plots, the latencies of approach (measured in seconds) during conspecific (dark gray), congeneric (black), and control (pale gray) trials are shorn. For each box plot, the total data range, the 25% and 75% quartiles (box), and the median are represented

In both congeneric pairs, we did not detect evidences of interspecific territoriality and males reached the closest distance from the speaker with the playbacks of conspecific males, staying equally further from congener and control songs (Figures 4 and 5; Appendix S8). Similarly, males approached the speaker faster when the playback broadcasted the song of a conspecific. Only the Ortolan bunting did not display differences in the latency between conspecific and congeneric songs (Figure 5; Appendix S8). However, its behavioral response to the congener was not different from that to the control, suggesting that latency may discriminate poorly in this species (Figure 5; Appendix S8). Contrarily to studies on species with heterospecific territorialism (Jankowski et al., 2010; Laiolo, 2013), we never observed the owner of the territory clearly approaching the speaker when the control or congener song was broadcasted, as they did when a conspecific song was broadcast (e.g., using the speaker as a post, performing short flights in and out the equipment). We checked whether our sample was large enough to detect a difference between the response to a congener and the control. For this, we hypothesized the response to congeners should be proportional to observed segregation between species (C‐scores), using the effect size of conspecific vs. control tests as the maximum possible response for territories that are fully defended (i.e., among homospecifics). As the C‐score of pipits 0.90 and 0.71 for buntings, the expected effect size for the heterospecific tests should be equal to the effect size between conspecific vs. control test (the maximum response for defended territories) multiplied by 0.90 (pipits) and 0.71 (buntings). We estimated the effect size for the response between conspecific and control according to Cohen (1992): Effect size = M1–M2/ Pooled standard deviation, where M1 is the mean of the response to the conspecific and M2 is the mean of the response to the control. The power for the comparison among heterospecifics, based on the sample size for this level and the correction of the effect size for homospecifics by the C‐score, was high. For the closest distance of approach, we obtained a power of 0.89 and 0.96 in Tree pipit and Water pipit, respectively. For the same variable, we obtained a power of 0.91 and 0.76 for Yellowhammer and Ortolan bunting. For the latency of approach, we obtained a power of 0.70 and 0.83 in pipits. For the same variable, we obtained a power of 0.88 and 0.83 in Yellowhammer and Ortolan bunting, respectively.

## 4 Discussion

Our study indicates that groups of species vary in how important environmental and biotic interactions are in driving their distributions. Teasing these factors apart is hard, and the combination of experimental and modeling approaches was crucial to sort among alternative hypotheses. The two pairs of congeneric species have a high level of local segregation, and in pipits, this is due to different environmental preferences and negative residual correlation between species, as highlighted by JSDM models (Figures 2 and 3). This supports the idea of competitive exclusion, although not mediated by direct agonistic interactions, as playback experiments revealed (Figure 4), although we cannot exclude that part of this residual correlation is reflecting some unmeasured environmental predictors. Conversely, segregation in buntings depends more on latitudinal and longitudinal partitioning than on environmental factors (Figures 2 and 3), and there is no evidence of interspecific aggressiveness in this species pair (Figure 5). The response of the species to a diverse set of factors appears therefore to be highly individualistic, even when the question is addressed in similar settings and when targeting species that are more likely to interact because of close phylogenetic similarity.

Pipits have a different thermal niche, the Tree pipit favoring the warmer conditions and the Water pipits the colder (Figure 2). They also partially segregate by habitat, selecting different kind of open lands (Figure 2). The spatial segregation of pipits does not appear to be a case of ecological character displacement (i.e., the results of past competition between species, Connell, 1980) or of allopatric speciation processes. Indeed although Tree and Water pipits are closest extant relatives, they are not sister species and their respective lineages have originated in different Palearctic zones, western for the species group including the Water pipit, and eastern for that of the Tree pipit (Voelker, 1999). Thus, the observed environmental differences might reflect the environmental conditions in which they evolved, and a process of niche tracking may underlie their current spatial distribution in these mountains (Laiolo, Seoane, Obeso, & Illera, 2017).

Apart from different environmental requirements, a crucial result of this study is that there is still a negative residual correlation between species (Figure 3). This provides some evidence that, at least in this congeneric pair, interspecific competition may also play a role in range partitioning at the local‐regional scale. The temperate latitudes and mountain conditions of this study may not preclude, therefore, negative interactions between congeneric species, as commonly observed in elevational replacement in the tropics (e.g., Jankowski et al., 2010), although these interactions are not mediated by interspecific territorialism. As pipits are responsive to conspecific territorial intrusions, we would have expected similar (or only slightly inferior) defense behavior with a heterospecific species if the observed spatial segregation between species is due also to interference mediated by interspecific aggressiveness. However, it must be acknowledged that field playback experiments cannot capture all of the possible forms of competition or even all the forms of interference. In fact, the lack of aggressiveness to congeneric territorial intrusion does not exclude alternative forms of negative interactions, like indirect exploitative competition driven by some limited food, or an avoidance mechanism such as individuals avoiding to settle in territories actively advertised by the congener (Smolla, Gilman, Galla, & Shultz, 2015). The above interference mechanism may work in pipits, because nonoverlapping territories are often relatively close, and may be even occupied by the two species in different periods of the breeding season (but never jointly; authors' pers. obs.). Observationally, we detected no aggressive response resembling the reaction to a conspecific even though we performed the experiments when territorial defense would have been maximal (Bastianelli et al., 2015). As territorial defense is a costly behavior (Orians & Willson, 1964), resource defense mediated by aggressiveness may become less profitable in seasonal environments, such as temperate mountains, where food resources are abundant but only for short periods (Minot & Perrins, 1986).

It is also possible that the negative residual correlation could be due to a missing environmental variable in JSDM, to which pipits respond differently (Pollock et al., 2014). Although the environmental predictors considered here have been shown to be important determinant of bird species distribution and species abundances in temperate environments (Chamberlain et al., 2016; Elsen et al., 2017; Meléndez & Laiolo, 2014; Seoane et al. 2017), we could not exclude the possible effect of unmeasured environmental variables. These may be some microclimatic variables not captured by the extrapolated digital layers of climate, or some fine measures of vegetation structure that could affect differently the two pipits. Negative residual correlation could also reflect other biotic factors like predator distributions and/or the distributions of other (not closely phylogenetically related) competitor species. However, predators are quite generalist in our study area and usually occupy a wide elevational gradient in the study area (Bastianelli et al., unpublished data). Moreover, Water and Tree pipit have a very similar territorial and breeding behavior (Bastianelli et al., 2015; Petrusková et al., 2014); thus, it is unlikely that some predator may affect just one of the two species up to excluding it from an entire elevation band. As Water and Tree pipits are each other's closest extant relatives in our study area (Laiolo et al., 2017), they are more likely to exhibit limiting similarity patterns than any other pair of species with which they coexists (Violle, Nemergut, Pu, & Jiang, 2011; Wiens et al., 2010). Competition is more likely among closely related species because these species display the strongest biological and ecological similarity (Elsen et al., 2017; Pigot et al. 2016).

Results obtained with buntings suggest a different scenario. Buntings share aspects of their ecological niche, as an example of conservation of the niche through species phylogeny. They co‐occur in some instances, and the residual correlation also is positive, though weak and uncertain (Figures 2 and 3). At a local scale, bunting distribution is better predicted by latitude and longitude than by climatic features (Appendix S1), which suggests a possible limitation to dispersal as a driver of partitioning in this pair of species (White, 2016). The presence of both increases in the southern slopes, although this effect is more marked in the Ortolan bunting (Figure 2). This species avoids the more northern slopes and settles preferentially in southwestern ones (Figure 2), where mountains have the lowest elevation and often do not reach 2,000 m a.s.l. This constrained distribution may be unexpected for a long‐distance migrant such as the Ortolan bunting, but the Cantabrian Mountains represent the northern distribution limits for several trans‐Saharian migratory birds in the Iberian Peninsula during reproductive season (e.g., White Stork, Whinchat, Bluethroat; Martí & Del Moral, 2003). Positive pairwise correlations, as between buntings—though uncertain, are not unusual among congeneric species in mountain chains (e.g., Himalaya; Elsen et al., 2017). Open questions for future and more direct studies are the occurrence of heterospecific attraction or facilitation processes (Mönkkönen et al., 1997; Sebastián‐González et al., 2010; Thomson, Forsman, & Mönkkönen, 2003).

The lack of generality of competition‐driven processes in determining range replacement is not typical of our study mountain system and has been observed in other mountainous contexts (Elsen et al., 2017). This heterogeneity in responses recalls Lewontin's (2002) claims on the multiple causes of evolutionary change; similarly, ecology is faced with many small contributing forces and teasing them apart is hard. The present study represents one of the first examples where joint species distribution modeling is combined with experimental evidence to tease apart the relative importance of biotic and abiotic distribution drivers at a fine scale. The combination of both methods has permitted the analysis of the dynamic of geographical partitioning, identifying the causal mechanisms that underlies the correlative patterns. In particular in the case of pipits, JSDM indicates that pipits may be competing when classic theory would suggest they should not be due to the extreme environment (although we could not completely exclude some unmeasured environmental driver). The experimental approach suggests that the aggressive behaviors commonly expressed during territorial disputes are not the means by which interspecific competition is mediated in this case. We recommend application of complementary approaches to inference, as implemented here, in order to deeply scrutinize causal drivers of species distributions.

## Author Contributions

Giulia Bastianelli and Paola Laiolo originally formulated the idea; Giulia Bastianelli, Javier Seoane, and Paola Laiolo designed the playback experiment and performed fieldwork; Giulia Bastianelli, Brendan A. Wintle, Elizabeth Hazel Martin, and Paola Laiolo formulated the statistical approach; Giulia Bastianelli and Elizabeth Hazel Martin performed statistical analyses; Giulia Bastianelli wrote the manuscript, and all the authors critically revised the manuscript.

## Conflict of Interest

None declared.
