# Supplementary material for: Species partitioning in a temperate mountain chain: Segregation by habitat vs. interspecific competition
Source: Ecol Evol. 2017 Mar 19;7(8):2685–96. doi: 10.1002/ece3.2883 (PMC5395447; doi:10.1002/ece3.2883)
Supplement: Supplementary file 1 [file ECE3-7-2685-s001.docx]

**Appendix S1.** Location of study area in Iberian Peninsula (a). In (b) and (c) the playback sites and bird survey plots in the Cantabrian Mountain for pipits and buntings are represented. The white-black colour gradient represents the Digital Elevation Model (DEM), darker areas indicate higher elevations. (b) Yellow arrows represent the playback areas; red dots represent the plots where the Water pipit is present; green dots represent the plots where the Tree pipit is present; blue dots represent the plots where both species are absent. (c) Yellow arrows represent the playback areas; green dots represent the plots where the Yellowhammer is present; orange dots represent the plots where the Ortolan bunting is present; blue dots represent the plots where both species are absent.

**
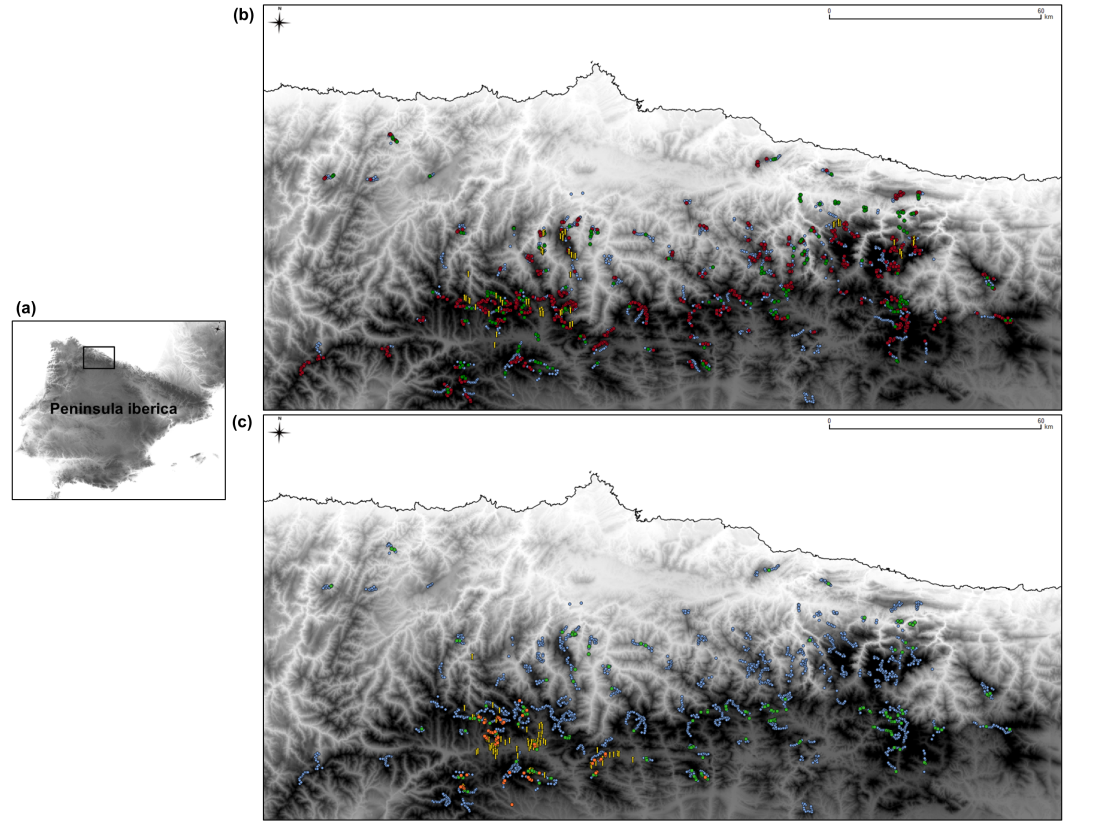
**

**Appendix S2.**  Selection of JSDM predictors. We inserted the predictors that explained at least 1% of deviance of the probability of presence in at least one of the two species of pair. We inserted a predictor as quadratic terms (calculating the orthogonal polynomials) when visual inspection suggested no-linear effects for some predictors in at least one of the two species of pair (we considered only quadratic effects because greater powers did not explain more substantial deviance than quadratic ones). In both pairs, annual mean temperature was highly and negatively correlated with elevation (pipits: r = - 0.97; buntings: r = - 0.81), thus we only considered the former in models, as the main climatic factor that varies uniformly along the elevational gradient. The predictors describing temperature condition (annual averages for the mean, maximum and minimum temperatures and temperature range) and those describing rainfall conditions (mean annual rainfall and rainfall range) were the only highly correlated (temperature predictors: R ≥ 0.76; rainfall predictors: R ≥ 0.65) and explained similar deviances thus we only considered mean annual temperature and rainfall in the JSDMs. Longitude and latitude were not correlated with any environmental factors, but they explained a large amount of deviance in the case of buntings and were thus entered in their JSDMs. Slope, solar radiation and roughness were considered as topographical predictors only for Buntings since they explained a deviance < 1 in Pipits. Microhabitat heterogeneity was not entered in any model because it explained a deviance < 1% for all the species.

**Selected predictors**

| **PREDICTOR** | **LINEAR EFFECT** | | **QUADRATIC EFFECT** | |
| --- | --- | --- | --- | --- |
|  | PIPITS | BUNTINGS | PIPITS | BUNTINGS |
| Mean annual temperature |  | X | X |  |
| Mean annual rainfall | X | X |  |  |
| Longitude |  | X |  |  |
| Latitude |  | X |  |  |
| Slope |  | X |  |  |
| Solar radiation | X | X |  |  |
| Roughness |  | X |  |  |
| Grass cover | X | X |  |  |
| High shrub cover |  |  | X |  |
| Low shrub cover | X |  |  |  |
| Tree cover | X | X |  |  |
| Rock cover |  | X | X |  |

**Appendix S6.** Playback experiments details. Number of tested males for each type of playback trials (a). Recording details (b). Experiment details (c).

**(a)**

|  | **Tree pipit** | **Water pipit** | **Yellow- hammer** | **Ortolan bunting** |
| --- | --- | --- | --- | --- |
| **Conspecific species** | 30 | 26 | 22 | 27 |
| **Congeneric species** | 25 | 22 | 16 | 16 |
| **Control species** | 25 | 20 | 16 | 15 |
| **Total** | 80 | 68 | 54 | 58 |

**(b)** We used a Marantz PMD661 recorder and a Sennheiser ME67 microphone to record song at a distance <20 m. The recordings were performed in good weather conditions to lower noise to signal ratio. Overall, we recorded at least 10 songs per individual male. We selected good quality songs, and overall we obtained 14.8 ± 2.32 SD individuals per species. Songs were filtered with a high pass filter at 0.8 KHz, to reduce background noise, and sound levels standardized to amplitude of 80% of a volt (Laiolo 2013, Bastianelli et al. 2015). The vocalization bouts were copied and appended several times to create a 4-minute broadcast consisting of repetitions of ten seconds of song and ten seconds of silence, reproducing as accurately as possible the song performances of each species.

**(c)** The songs were broadcast with a Marantz PMD661 recorder and Philips SBA1500/37 speaker placed close to individual posts (a rock or a bush). The movements and vocal activity of the territory owner were surveyed from a partially hidden spot located > 30 m away from the territory tested, observations being made with 10×40 binoculars by two observers at a time. This double control allowed to better track the movements of the focal (unmarked) individual, and excluded the possible confusion with other neighbouring individuals. The closest distance approached by the focal bird was established by means of a laser rangefinder, the latency times was measured (in seconds) by means of a stop watch. When birds did not approach the speaker, the times to approach were set to 480 s (the overall duration of the experimental observation; i.e. playback and post-playback phase), and their distance was recorded at the end of the experiment.

REFERENCES

Bastianelli, G. et al. 2015. The intensity of male-male interactions declines in highland songbird populations. - Behav. Ecol. Sociobiol. 69: 1493–1500.

Laiolo, P. 2013. From inter-specific behavioural interactions to species distribution patterns along gradients of habitat heterogeneity. - Oecologia 171: 207–215.

**Appendix S7.** Set of alternative joint species distribution models built with the criteria in Appendix S2 (a) for pipits and (b) for buntings. In each model, AUC mean (± SD) is shown for each species of the pair. The highest posterior density means (HPD) of both environmental and residual correlations, with their lower (2.5%) and upper (97.5%) credible intervals (CI), are shown for each model. Each model is built considering only the predictors explaining at least 1% of the deviance (T = Mean annual temperature; RF = Annual accumulated precipitation; LAT = Latitude; LONG = Longitude; SLO = Slope; RAD = Solar radiation; ROU = Roughness; G = % cover of grassland; HS = % cover of high shrubs; LS = % cover of low shrubs; TR = % cover of trees; RO = % cover of rocks).

**(a)**

| **Model** | **AUC** | | **Environmental**  **correlation** | **Residual**  **correlation** |
| --- | --- | --- | --- | --- |
|  | **Tree pipit** | **Water pipit** | **HPD** | **HPD** |
| T^2^ RF RAD G HS^2^ LS TR RO^2^ | 0.75 ± 0.02 | 0.83 ± 0.01 | -0.54 (CI:-0.92/-0.02) | -0.37 (CI:-0.50/-0.24) |
| G HS^2^ LS TR RO^2^ | 0.73 ± 0.03 | 0.81 ± 0.02 | -0.36 (CI:-0.91/0.28) | -0.41 (CI:-0.54/-0.28) |
| T RF RAD G HS LS TR RO | 0.74 ± 0.04 | 0.79 ± 0.02 | -0.49 (CI:-0.61/-0.37) | -0.40 (CI:-0.52/-0.26) |
| T^2^ RF RAD | 0.67 ± 0.01 | 0.74 ± 0.02 | -0.90 (CI:-0.99/-0.80) | -0.43 (CI:-0.54/-0.32) |
| T^2^ RF | 0.67 ± 0.01 | 0.74 ± 0.02 | -0.90 (CI:-0.99/-0.79) | -0.42 (CI:-0.53/-0.31) |

**(b)**

| **Model** | **AUC** | | **Environmental**  **correlation** | **Residual**  **correlation** |
| --- | --- | --- | --- | --- |
|  | **Yellow hammer** | **Ortolan bunting** | **HPD** | **HPD** |
| LONG LAT RAD ROU G TR RO | 0.80 ± 0.06 | 0.91 ± 0.01 | 0.55 (CI: 0.35/0.73) | 0.17 (CI:-0.06/0.40) |
| LONG LAT SLO ROU G TR RO | 0.79 ± 0.05 | 0.91 ± 0.02 | 0.56 (CI: 0.36/0.74) | 0.18 (CI: -0.06/0.42) |
| LONG LAT SLO RAD ROU | 0.69 ± 0.08 | 0.91 ± 0.02 | 0.52 (CI:0.28/0.73) | 0.22(CI: -0.001/0.43) |
| T RF LONG LAT SLO ROU RAD | 0.78 ± 0.06 | 0.90 ± 0.02 | 0.36 (CI:0.16/0.55) | 0.27(CI:0.04/0.47) |
| LONG LAT | 0.62 ± 0.06 | 0.87 ± 0.02 | 0.32 (CI: -0.19/0.75) | 0.31(CI: 0.11/0.50) |
| T RF SLO ROU G TR RO | 0.78 ±0.04 | 0.83 ± 0.05 | 0.67 (CI: 0.45/0.84) | 0.19(CI: -0.02/0.39) |
| SLO RAD ROU G TR RO | 0.78 ± 0.04 | 0.80 ± 0.05 | 0.76 (CI: 0.52/0.92) | 0.13(CI:-0.08/0.32) |
| T RF RAD ROU G TR RO | 0.77 ± 0.04 | 0.83 ± 0.03 | 0.62 (CI: 0.40/0.81) | 0.21(CI: 0.01/0.41) |
| G TR RO | 0.77 ± 0.06 | 0.75 ± 0.04 | 0.85 (CI: 0.60/0.98) | 0.16(CI: -0.04/0.34) |
| T RF SLO ROU RAD | 0.70 ± 0.05 | 0.78 ± 0.05 | 0.39(CI: 0.11/0.64) | 0.28(CI: 0.08/0.47) |
| T RF | 0.64 ± 0.04 | 0.63 ± 0.05 | -0.64(CI:-0.99/-0.01) | 0.35(CI: 0.17/0.52) |

**Appendix S8.**

Table of results of one-way ANOVA, Kruskal-Wallis test and relative multiple pairwise comparisons testing for the effect of three playback types (conspecific, congeneric and control) on the minimum distance of approach (a) and on the latency of the approach (b) during playback trials. (a)

|  |  | ***Minimum distance of approach*** | |  |  |  |  |  |  |  |
| --- | --- | --- | --- | --- | --- | --- | --- | --- | --- | --- |
|  |  |  |  |  |  |  |  |  |  |  |
| **Genus** | **Species** | **One-way Anova** | *df* | *F* | *p* | **Multiple comparisons** | Estimate | SE | t | *p* |
|  | Tree pipit | Treatment | 2 | 7.39 | 0.001 | congeneric-conspecific | 0.77 | 0.27 | 2.87 | 0.01 |
|  |  | Residuals | 77 |  |  | control-conspecific | 0.90 | 0.26 | 3.40 | 0.003 |
| ***Anthus*** |  |  |  |  |  | control-congeneric | 0.12 | 0.25 | 0.50 | 0.87 |
|  | Water pipit |  |  |  |  |  |  |  |  |  |
|  |  | Treatment | 2 | 20.75 | <0.001 | congeneric-conspecific | 1.32 | 0.21 | 6.18 | < 0.001 |
|  |  | Residuals | 65 |  |  | control-conspecific | 0.94 | 0.24 | 3.93 | < 0.001 |
|  |  |  |  |  |  | control-congeneric | -0.37 | 0.19 | 1.95 | 0.13 |
|  | Yellowhammer |  |  |  |  |  |  |  |  |  |
|  |  | Treatment | 2 | 16.29 | <0.001 | congeneric-conspecific | 1.46 | 0.31 | 4.72 | < 0.001 |
|  |  | Residuals | 51 |  |  | control-conspecific | 1.58 | 0.34 | 4.61 | < 0.001 |
| ***Emberiza*** |  |  |  |  |  | control-congeneric | 0.12 | 0.27 | 0.45 | 0.89 |
|  | Ortolan bunting |  |  |  |  |  |  |  |  |  |
|  |  | Treatment | 2 | 8.64 | <0.001 | congeneric-conspecific | 2.55 | 0.84 | 3.03 | 0.01 |
|  |  | Residuals | 55 |  |  | control-conspecific | 3.14 | 0.80 | 3.92 | < 0.001 |
|  |  |  |  |  |  | control-congeneric | 0.60 | 0.79 | 0.76 | 0.73 |

(b)

|  |  | ***Latency of approach*** |  |  |  |  |  |  |
| --- | --- | --- | --- | --- | --- | --- | --- | --- |
|  |  |  |  |  |  |  |  |  |
| **Genus** | **Species** | **Kruskal -Wallis test** | *df* | *χ²* | *p* | **Multiple comparisons** | z | *p* |
|  | Tree pipit | Treatment | 2 | 8.66 | 0.01 | conspecific-congeneric | -2.58 | 0.03 |
|  |  |  |  |  |  | conspecific-control | -2.44 | 0.04 |
| ***Anthus*** |  |  |  |  |  | congeneric-control | 0.13 | 1 |
|  | Water pipit |  |  |  |  |  |  |  |
|  |  | Treatment | 2 | 13.08 | 0.001 | conspecific-congeneric | -3.22 | 0.004 |
|  |  |  |  |  |  | conspecific-control | -2.92 | 0.01 |
|  | Yellowhammer |  |  |  |  | congeneric-control | 0.21 | 1 |
|  |  |  |  |  |  |  |  |  |
|  |  | Treatment | 2 | 18.82 | <0.001 | conspecific-congeneric | -3.78 | <0.001 |
|  |  |  |  |  |  | conspecific-control | -3.53 | 0.001 |
| ***Emberiza*** |  |  |  |  |  | congeneric-control | 0.23 | 1 |
|  | Ortolan bunting |  |  |  |  |  |  |  |
|  |  | Treatment | 2 | 12.47 | 0.002 | conspecific-congeneric | -2.1 | 0.11 |
|  |  |  |  |  |  | conspecific-control | -3.41 | 0.002 |
|  |  |  |  |  |  | congeneric-control | -1.21 | 0.68 |
